# Molecular Cocrystals with Hydrogen-Bonded Polymeric Structures and Polarized Luminescence

**DOI:** 10.3390/ma15207247

**Published:** 2022-10-17

**Authors:** Jing-Yi Zhao, Fa-Feng Xu, Zhong-Qiu Li, Zhong-Liang Gong, Yu-Wu Zhong, Jiannian Yao

**Affiliations:** 1 Beijing National Laboratory for Molecular Sciences, CAS Key Laboratory of Photochemistry, CAS Research/Education Center for Excellence in Molecular Sciences, Institute of Chemistry, Chinese Academy of Sciences, Beijing 100190, China; 2 School of Chemical Sciences, University of Chinese Academy of Sciences, Beijing 100049, China

**Keywords:** molecular crystals, hydrogen bond, luminescence, assembly

## Abstract

Crystalline materials with appealing luminescent properties are attractive materials for various optoelectronic applications. The in situ bicomponent reaction of 1,2-ethylenedisulfonic acid with 1,4-di(pyrid-2-yl)benzene, 1,4-di(pyrid-3-yl)benzene, or 1,4-di(pyrid-4-yl)benzene affords luminescent crystals with hydrogen-bonded polymeric structures. Variations in the positions of the pyridine nitrogen atoms lead to alternating polymeric structures with either a ladder- or zigzag-type of molecular arrangement. By using a nanoprecipitation method, microcrystals of these polymeric structures are prepared, showing polarized luminescence with a moderate degree of polarization.

## 1. Introduction

Micro/nanoscale assembly materials with polarized luminescence have attracted great attention in fields of anisotropic emission [1,2,3,4], circular polarized luminescence [5,6,7], lasers [8,9], etc. By means of aligning small molecules into ordered arrangements with noncovalent intermolecular interactions, various organic micro/nanocrystalline assemblies have been developed [10,11,12]. Among them, hydrogen bonds featuring moderate interaction strength and high directionality have been employed in bottom-up assemblies [13,14]. Furthermore, multicomponent coassembly relying on hydrogen-bonding interactions has been demonstrated as an effective strategy for the construction of low-dimensional organic microcrystals and micro/nanostructures [15,16]. However, the efficient preparation and rational manipulation of multicomponent molecular cocrystals at micro/nanoscales is still a challenging task.

Pyridine-containing functional chromophores are important organic dyes, which exhibit interesting acid/base stimuli-responsive emission characteristics in solutions [17,18] and the crystal state [19,20,21,22,23,24]. The multicomponent assemblies of pyridine-containing chromophores have become a versatile platform to prepare molecular nanostructures with appealing photonic applications in recent decades [25,26,27,28]. Here, we present a simple strategy for the fabrication of hydrogen-bonded microscale molecular cocrystals using the in situ coassembly of linear chromophores containing two terminal pyridine groups with a linear disulfonic acid component. Cocrystals are a common and useful strategy for creating ordered and crystalline materials from an assembly of multiple components. For instance, the formation of cocrystals is an effective means for enhancing the solubility and release dynamics of some drugs [29,30,31]. In addition, the pyridine–carboxylic acid interaction is commonly employed in crystal engineering to obtain mono- or multicomponent crystals [32,33,34,35]. However, only limited numbers of hydrogen-bonded cocrystals have been reported to show emission properties toward photonic applications [28,36].

**Figure 1:**
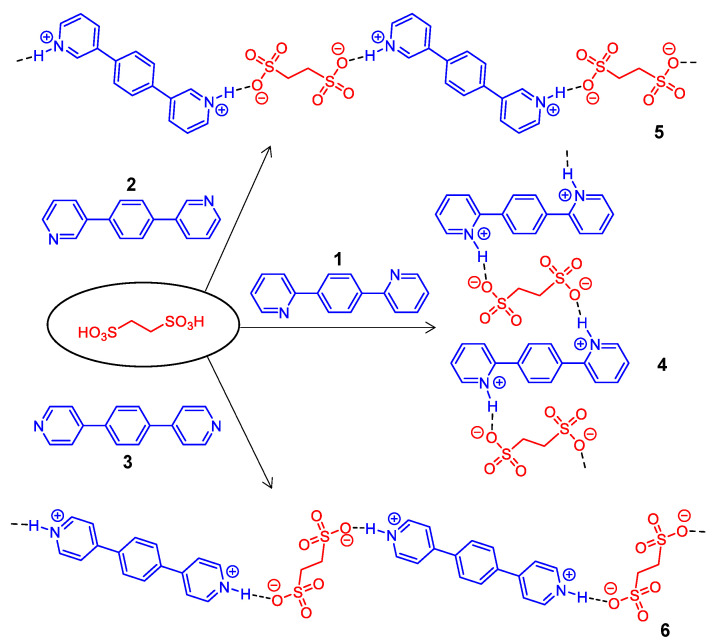
Schematic diagram of the reaction of 1,2-ethylenedisulfonic acid with **1**–**3** to give polymeric structures **4**–**6**, respectively.

Our recent works show that the in situ reaction and crystallization of a mixture of pyridine-functionalized organic chromophores with a strong protonic acid, such as sulfonic acid and perchloric acid, are able to provide cocrystals with an ordered morphology and intense emission [4,16]. The use of a strong acid in this method, instead of a common carboxylic acid compound, leads to the protonation of the pyridine units to give ionic pyridinium moieties with strong charge transfer emissions. Stimulated by these results, we are interested to examine the potential of the reactions of dipyridine-functionalized organic chromophores with a diacid component. Specifically, the in situ reaction of the pyridine-containing molecule 1,4-di(pyrid-2-yl)benzene (**1**), 1,4-di(pyrid-3-yl)benzene (**2**), or 1,4-di(pyrid-4-yl)benzene (**3**) with 1,2-ethylenedisulfonic acid was found to yield protonated pyridinium-sulfonate binary cocrystals **4**–**6**, respectively, with hydrogen-bonded polymeric structures (Figure 1). Molecules **1**–**3** with a 1,4-dipyridylbenzene skeleton are commonly used as ligands in coordination chemistry [37,38]. They were selected as the model compounds for the binary assembly with 1,2-ethylenedisulfonic acid in the hope of tuning the structures and properties of the obtained cocrystals through engineering the hydrogen-bond directions. The variation of the positions of the pyridine nitrogen atoms on the skeleton allows for the formation of hydrogen bonds between pyridinium protons and sulfonate anions in different directions. In addition, the polarized emission properties of organic crystals are highly dependent on the molecular arrangement, and those with consistent parallel or antiparallel molecular orientation generally display a high degree of emission polarization [39,40,41,42]. On account of the highly ordered binary molecular packing in two-component linear hydrogen-bonded polymers, the fabricated microcrystals are expected to display prominent polarized emissions.

## 2. Materials and Methods

### 2.1. Materials

Compounds 1,4-di(pyrid-2-yl)benzene (**1**), 1,4-di(pyrid-3-yl)benzene (**2**), and 1,4-di(pyrid-4-yl)benzene (**3**) were purchased from Nanchang Chouhechem Pharmatech Co., Ltd., (Nanchang, China) and used as received without further treatment. The compound 1,2-ethylenedisulfonic acid hydrate was obtained from Anhui Senrise Technology Co., Ltd. (Anqing, Anhui Province, China). The solvent of methanol used in this article was chromatographically pure.

### 2.2. Sample Preparation

Growth of Single Crystals of **4**, **5**, and **6**. These single crystals were obtained via slow solvent evaporation from a mixed solution of 1,2-ethylenedisulfonic acid with **1** (1.2 mM), **2** (0.4 mM), or **3** (0.1 mM), respectively, in methanol in a 1:1 molar ratio.

Preparation of Microcrystals of **4**. The microcrystals were prepared with a nanoprecipitation method. A solution of **1** (1 mL, 20 mM) in MeOH was quickly injected into a solution of 1,2-ethylenedisulfonic acid (1 mL, 20 mM) in MeOH. After mild ultrasonication for 2 min, a colloid solution was obtained, which was aged for 2 h at rt to give a mixture containing the microcrystals of **4** at the bottom of the vial. After filtration, 3.7 mg of **4** was obtained in 43.8% yield. ^1^ H NMR (400 MHz, DMSO-d_6_) δ 8.75 (d, J = 4.2 Hz, 2H), 8.24 (s, 4H), 8.16 (d, J = 7.9 Hz, 2H), 8.07 (t, J = 7.4 Hz, 2H), 7.56–7.48 (m, 2H), 2.64 (s, 4H).

Preparation of Microcrystals of **5**. Using the same method for the preparation of the microcrystals of **4**, those of **5** were obtained from **2** (1 mL, 20 mM) and 1,2-ethylenedisulfonic acid (1 mL, 20 mM) in 69.9% yield (5.9 mg of 5 was obtained). ^1^H NMR (400 MHz, DMSO-*d*_6_) δ 9.12 (s, 2H), 8.73 (d, *J* = 5.0 Hz, 2H), 8.46 (d, *J* = 7.7 Hz, 2H), 7.98 (s, 4H), 7.76 (dd, *J* = 7.7, 5.3 Hz, 2H), 2.63 (s, 4H).

Preparation of Microcrystals of **6**. Using the same method for the preparation of the microcrystals of **4**, those of **6** were obtained from **3** (1 mL, 20 mM) with 1,2-ethylenedisulfonic acid (1 mL, 20 mM) in a 71.1% yield (6.0 mg of 6 was obtained). ^1^H NMR (400 MHz, DMSO-*d*_6_) δ 8.80 (d, *J* = 6.1 Hz, 4H), 8.09 (s, 4H), 8.05 (s, 4H), 2.61 (s, 4H).

### 2.3. Characterization

X-Ray Diffraction Analysis. The single-crystal X-ray diffraction data were collected using a Talab Synergy-R diffractometer on a rotating anode (Cu Kα radiation, 1.54184 Å) at 170 K. The structure was solved with the direct method using SHELXS-97 and refined with Olex 2. CCDC numbers were 2181166, 2208263, and 2181168 for crystals of **4**, **5**, and **6**, respectively. Powder X-ray diffractions were carried out on a Malvern Panalytical Empyrean instrument.

### 2.4. Equipment Information of Physical Measurements

^1^H NMR spectra were recorded on a Bruker Avance 400 MHz spectrometer. Diffusion-ordered spectroscopy (DOSY) NMR data were acquired with a Bruker Avance 600 MHz spectrometer. FTIR spectra were obtained on a Bruker VERTEX 70 v spectrometer. A thermogravimetric analysis (TGA) experiment was carried out on a Diamond TG/DTA analyzer from PerkinElmer Inc. The photoluminescence and UV/VIS absorption spectrum at room temperature were measured with an F-380 spectrofluorimeter from Tianjin Gangdong Sci. & Tech. Development Co., Ltd., (Tianjin, China) and a PerkinElmer UV/VIS/NIR spectrometer Lambda 750 with a 150 mm integrating sphere, respectively. Bright-field and fluorescence microscopy characterization was carried out using an Olympus BX53M microscope by exciting the samples with LED and mercury lamps, respectively. The morphology and crystallinity of microcrystals were examined with SEM using a Hitachi SU8010 instrument operating at 10 kV. The absolute emission quantum yield and excited-state emission lifetimes were measured with a Hamamatsu Quantaurus-QY spectrometer C11347 and Hamamatsu Quantaurus-Tau spectrometer C11367, respectively. The polarized luminescence measurements for individual microcrystals were carried out on a custom micro-photoluminescence system, as illustrated in Supplementary Materials Figure S12.

### 2.5. Methods of Calculation

The electronic structures of [**1**-H_2_]^2+^, [**2**-H_2_]^2+^, and [**3**-H_2_]^2+^ were optimized by using the density functional theory (DFT) calculations on the Gaussian 09 program package with the B3LYP exchange correlation functional and the 6–31 G ** basis set [43]. Solvent effects in CH_3_OH were included. Time-dependent DFT (TDDFT) calculations were performed on the optimized structures with the same level of theory.

## 3. Results

### 3.1. Studies on Single Crystals

Single crystals of the hydrogen-bonded binary cocrystals of **4**, **5**, and **6** were obtained via a direct evaporation method from the stock solution of a mixture of 1,2-ethylenedisulfonic acid with **1**, **2**, and **3** (1:1 molar ratio; 0.1–1.2 mM in CH_3_OH), respectively, under ambient conditions. X-ray diffraction (XRD) data of these single crystals showed that the crystals of **4** and **5** belonged to crystallographic triclinic groups and the crystal of **6** had a monoclinic group. The crystallographic data are summarized in Table 1.

**Figure 2:**
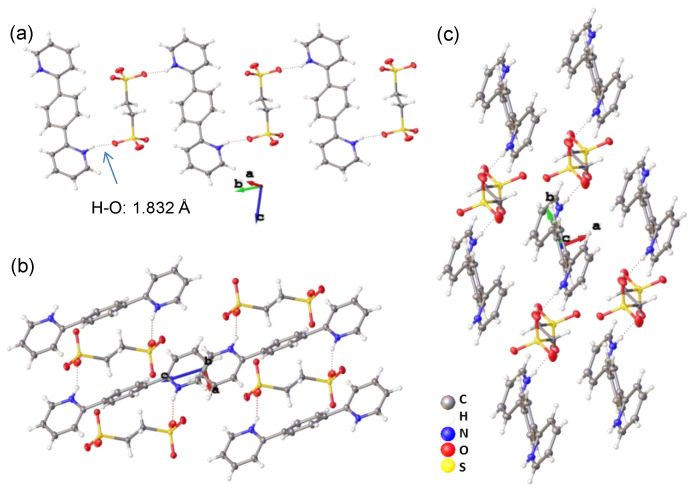
Molecular packing of the single crystal of **4** viewed from the (**a**) *a*, (**b**) *b*, and (**c**) *c* axes.

**Figure 3:**
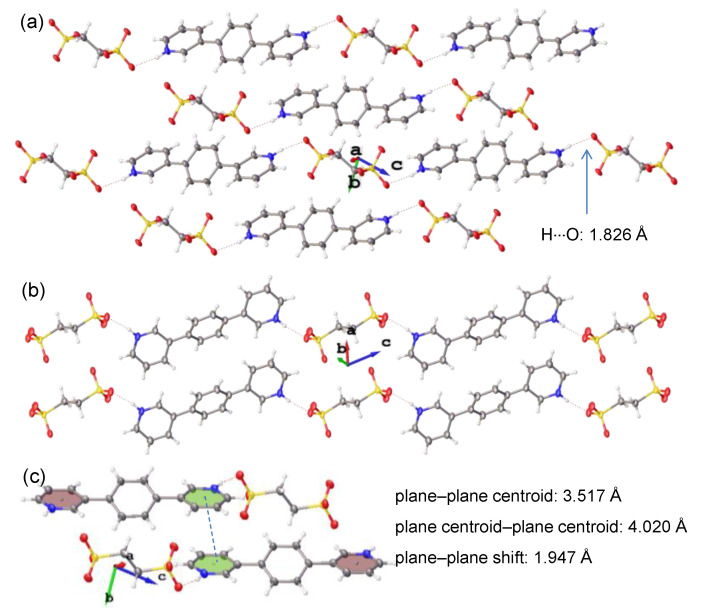
(**a**,**b**) Molecular packing of the single crystal of **5** viewed from the (**a**) *a* and (**b**) *b* axes, respectively. (**c**) Molecular stacking with potential π···π interaction of **5**.

**Figure 4:**
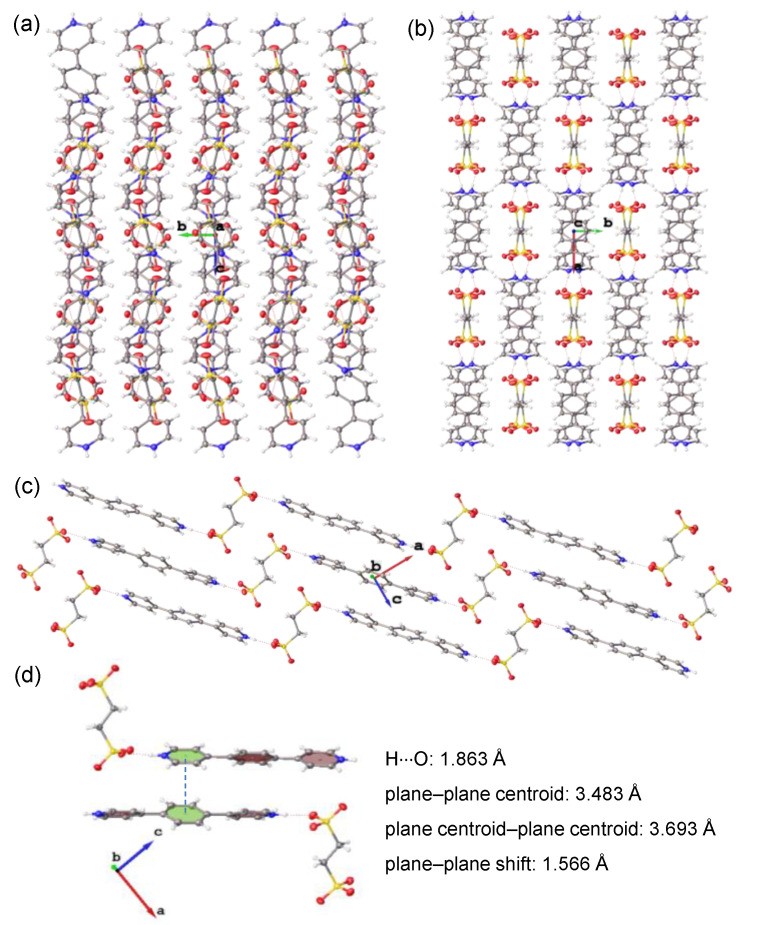
(**a**–**c**) Molecular packing of the single crystal of **6** viewed from the (**a**) *a*, (**b**) *c*, and (**c**) *b* axis, respectively. (**d**) Molecular stacking with potential π···π interaction of **6**.

The molecular packings of the single crystals of **4**–**6** are displayed in Figure 2, Figure 3 and Figure 4. The pyridine groups of the parent molecules (**1**–**3**) were protonated with acid to give ionic pyridinium structures. In the resulting crystals of **4**–**6**, there were hydrogen bonding interactions between the pyridinium hydrogen atoms and the anionic sulfonate oxygen atoms, with H∙∙∙O lengths of 1.832 Å (**4**), 1.826 Å (**5**), and 1.863 Å (**6**), respectively. Because each molecule of **1**–**3** contained two terminal pyridine groups, and 1,2-ethylenedisulfonic acid possessed two terminal sulfonic acid units, their alternating bicomponent reactions gave rise to hydrogen-bonded polymeric structures. However, as the positions of the pyridine nitrogen atoms changed, the obtained crystals of **4**–**6** displayed different polymeric configurations and molecular packing.

The bispyridinium and bis-sulfonate molecules of **4** were arranged in an alternating and parallel fashion to give a hydrogen-bonded polymer with a ladder-type configuration (Figure 2a). The molecular orientations of the long axes of both bispyridinium and bis-sulfonate molecules were nearly parallel to the *c* axis of the crystal cell (Figure 2b). As viewed from the *c* axis, the bispyridinium and bis-sulfonate molecules were well separated from the surrounding structures (Figure 2c). The shortest interplanar distance between adjacent pyridine/benzene segments was longer than 5 Å, suggesting that no distinct π···π interactions were present in crystal **4**.

In the crystal of **5**, the hydrogen-bonded polymer took an alternating zigzag shape, in which the bispyridinium and bis-sulfonate molecules were parallel to each other and their long axes were aligned in the same direction as the *c* axis of the crystal cell (Figure 3b). Some weak π···π interactions were present between the pyridinium units of adjacent polymeric structures, as shown in Figure 3c. The polymeric structure of **6** also displayed an alternating zigzag shape (Figure 4c). However, the long axes of the bispyridinium and bis-sulfonate molecules were almost perpendicular to each other, which was somewhat different with respect to those of **5**. The molecular packing showed that the polymeric chains of **6** were packed in a distinct layered arrangement, as viewed from the *a* axis of the crystal cell (Figure 4a). The image shown in Figure 4c corresponds to the top view of a single-layer structure, in which the polymeric chains were stacked compactly with the presence of π···π interactions between interchain benzene and pyridinium moieties (Figure 4d).

### 3.2. Studies of Microcrystals

**Figure 5:**
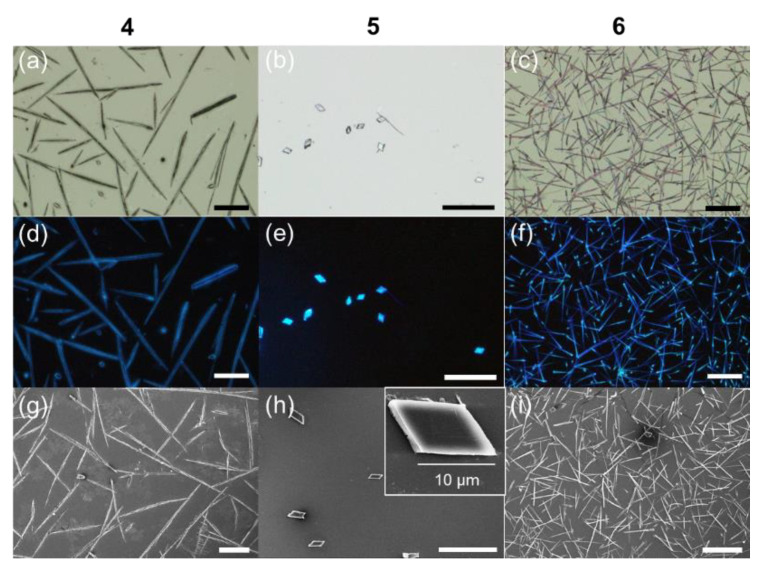
(**a**–**c**) Bright-field and (**d**–**f**) fluorescent microscopy images and (**g**–**i**) SEM images of the microcrystals of (**a**,**d**,**g**) **4**, (**b**,**e**,**h**) **5**, and (**c**,**f**,**i**) **6**. The fluorescent microscopy images were captured under the illumination of UV band (330–385 nm) of a mercury lamp. The inset of panel (**h**) shows an enlarged crystal. Scale bars are 50 μm unless otherwise noted.

In conjugation with the recent interest in organic nanophotonics on the basis of luminescent nano/microcrystals [9,10,11,12], we intended to obtain small-sized crystals from the bicomponent reactions of 1,2-ethylenedisulfonic acid with **1**–**3**, respectively. By using a nanoprecipitation method, in which a concentrated stock solution of **1**, **2**, or **3** (20 mM in CH_3_OH) was quickly injected into the solution of 1,2-ethylenedisulfonic acid (20 mM in CH_3_OH) under mild sonification, we were able to prepare microcrystals of **4** and **6** with a rod- or needle-like shape and that of **5** with a platelet shape (Figure 5). The microrods of **4** had sizes of 50–100 μm in length and 3−5 μm in diameter. The platelet microcrystals of **5** showed a rhomboid morphology with a length of 8–15 μm and thickness of approximately 1 μm, as revealed by using the scanning electronic microscopy (SEM) analysis (Figure 5h). The microneedles of **6** had lengths of 30–80 μm and diameters of 1–2 μm. These microcrystals displayed bright deep-blue or cyan emissions under the illumination of a mercury lamp (Figure 5d–f).

^1^H NMR spectra of these microcrystals were recorded in deuterated dimethyl sulfoxide (DMSO-*d*_6_; Figures S1, S3, and S5). The ^1^H signals from both the phenyl-bridged bipyridinium moiety and the ethylene unit of 1,2-ethylenedisulfonate could be discerned. The integration of the ^1^H signals suggested that these two components were present in a 1:1 molar ratio in the microcrystals. However, the hydrogen-bonded polymeric structures may not have been present in the solution, which would have been disrupted by the DMSO-*d*_6_ solvent. This was supported by the 2D DOSY NMR spectra of these crystals (Figures S2, S4 and S6). These ^1^H NMR signals displayed a diffusion coefficient of 2.031 × 10^−10^, 2.063 × 10^−10^, and 2.177 × 10^−10^ m^2^/s for **4**–**6**, respectively, in accordance with typical values for small molecules. The FTIR spectra of these microcrystals in KBr pellets showed the presence of broad peaks at approximately 3500 cm^−1^, which could be attributed to the vibrations of hydrogen bonds (Figure S7). The TGA results showed that the decomposition temperatures of **4**–**6** were 298, 240, and 311 °C, respectively (Figure S8).

**Figure 6:**
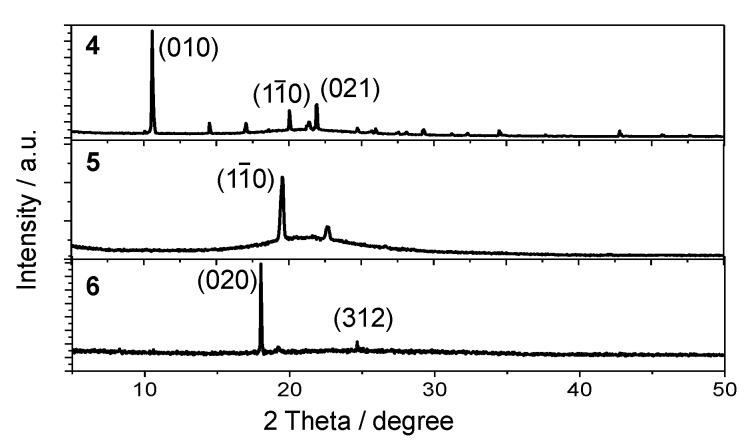
PXRD data of microcrystals of **4**–**6**.

The powder X-ray diffraction (PXRD) results illustrated the crystallinity and regularity of the above microcrystals (Figure 6). A set of prominent diffraction peaks was observed, indexed to the exposed crystallographic planes of (010) of **4**, (1–10) of **5**, and (020) of **6**, respectively, besides some weak higher-order peaks. These peaks were in accordance with the simulated patterns of corresponding single-crystal diffraction data (Figure S9).

**Figure 7:**
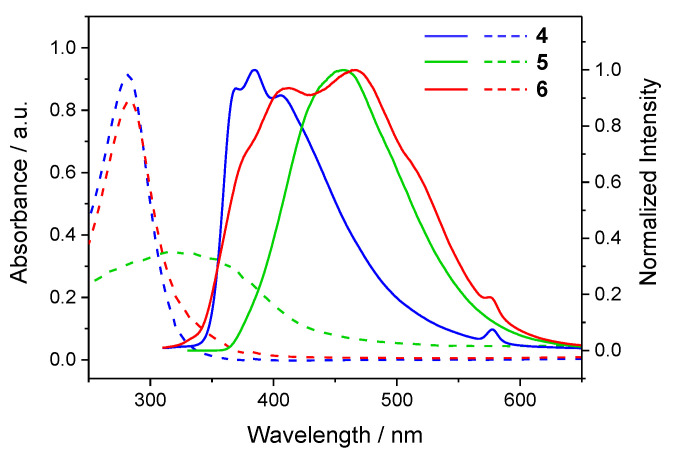
Absorption (dashed lines) and photoluminescence (solid lines) spectra of microcrystals of **4**–**6**. Excitation wavelength: 280 nm for **4** and **6**; 315 nm for **5**.

Figure 7 displays the absorption and emission spectra of the microcrystals of **4**–**6**. Their absorption spectra were mainly located in the UV band range with the maximum absorption wavelength at 280, 315, and 282 nm, respectively. In particular, the microcrystals of **5** showed much shallower and broader absorption with respect to those of **4** and **6**. Accordingly, the emission spectrum of **5** was significantly red-shifted with respect to that of **4**, with an emission maximum wavelength at 385 and 456 nm, respectively. The emission spectrum of **6** covered a broader wavelength range, between 350 and 600 nm, with respect to those of **4** and **5**. The reason for these differences was not clear at this stage. The π···π interactions in the crystals of **5** and **6** may have played a role. The absolute emission quantum yields of the microcrystals of **4**–**6** were 12.9%, 27.7%, and 18.0%, respectively. Their photoluminescence lifetimes were in the range of 1.8–11.7 ns (Table S1 and Figure S10).

The absorption and emission properties of **4**–**6** are believed to be largely determined by the π-conjugated phenyl-bipyridinium moiety. DFT and TDDFT calculations were performed on the bisprotonated forms of **1**–**3**, namely, [**1**-H_2_]^2+^, [**2**-H_2_]^2+^, and [**3**-H_2_]^2+^, which were taken as the model compounds for the polymeric structures of **4**–**6**, respectively (Figure S11). In the case of [**1**-H_2_]^2+^, both the highest occupied molecule orbital (HOMO) and lowest unoccupied molecular orbital (LUMO) had delocalized structures across the whole phenyl-bipyridinium moiety, suggesting that the HOMO → LUMO transition had a π/π* character. However, in the cases of [**2**-H_2_]^2+^ and [**3**-H_2_]^2+^, heavy contributions of the charger transfer from the central phenyl unit to the terminal pyridinium groups were involved. These results suggested that the emission of **4** had a major π/π* character, while those of **5** and **6** were dominated by charger transfer processes.

### 3.3. Polarized Luminescence

**Figure 8:**
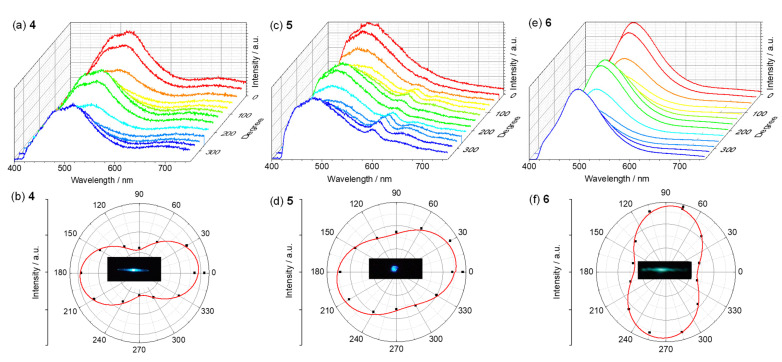
(**a**,**c**,**e**) Photoluminescent spectra and (**b**,**d**,**f**) corresponding polarization angle-dependent luminescence intensity distribution curves of a microcrystal of (**a**,**b**) **4**, (**c**,**d**) **5**, and (**e**,**f**) **6**. The red solid lines in panels (**b**,**d**,**f**) are fitted curves with the mathematical function of sin^2^. Insets are the fluorescent microscopy images of corresponding microcrystals. All microcrystals were excited with a focused semiconductor laser (CW, 405 nm).

Micro/nanoscale crystalline materials with polarized emissions are important functional materials for nanophotonic applications [11,44]. The polarized emission characteristics of single microcrystals were determined on a home-made luminescence microscopy platform (Figure S12). The microcrystal was excited with a 405 nm continuous-wave (CW) laser. The photoluminescence intensity of the microcrystal displayed an angle-dependent modulation of emission intensity as the polarization analyzer in front of the spectrometer was rotated to a certain degree (Figure 8). Accordingly, the corresponding polarization emission profile yielded a fitted curve in the shape of the number “8”. The long axis of the fitted polarization emission profile was in accordance with the oriented direction of the molecular transition dipoles (*μ*) of the crystal under study [39,40,41,42]. In other words, when the electric field of the light was parallel to *μ*, the emission was the highest (*I*_max_), while the emission was the lowest (*I*_min_) when the electric field was vertical to *μ*. The property of polarization emission could be evaluated with the polarization degree *P*, which was determined by using *P* = (*I*_max_ − *I*_min_)/(*I*_max_ + *I*_min_). The microcrystals of **4**–**6** were estimated to have *P* of 0.44, 0.24, and 0.35, respectively. This reflected the moderate polarized emission property of these crystals. Organic and metal–organic microcrystals possessing larger *P* values of approximately 0.9 have been reported [3,4]. In addition to the molecular orientation, the *P* value was highly dependent on the quality of the crystal. The moderate *P* values of **4**–**6** were possibly caused by the relatively low quality of these crystals.

## 4. Conclusions

In summary, luminescent crystalline materials were prepared from the bicomponent reaction of linear dipyridine molecules with 1,2-ethylenedisulfonic acid. These crystals possessed polymeric molecular structures linked by hydrogen bonds between pyridinium protons and sulfonate oxygen atoms. Corresponding microcrystals displayed polarized emission characteristics with a moderate degree of polarization. The changes of the positions of the pyridine nitrogen atoms led to hydrogen-bonded polymeric structures with different (ladder- or zigzag-type) molecular arrangements. We are currently working on the expansion of this bicomponent reaction method into other pyridine-functionalized chromophores in the hope of obtaining nano/microcrystals with improved luminescent properties.

## Figures and Tables

**Table 1:** Crystallographic data of **4**–**6**.

Compound	4	5	6
CCDC number	2181166	2208263	2181168
empirical formula	C_18_H_18_N_2_O_6_S_2_	C_18_H_18_N_2_O_6_S_2_	C_18_H_18_N_2_O_6_S_2_
formula weight	422.46	422.46	422.46
temperature (K)	170.00(10)	170.00(10)	169.99(10)
crystal system	Triclinic	Triclinic	Monoclinic
space group	*P*-1	*P*-1	*P*2/*c*
a (Å)	5.18630(10)	5.4682(3)	16.0369(2)
b (Å)	8.3578(2)	8.5731(4)	9.90610(10)
c (Å)	10.4965(2)	9.9083(5)	11.22670(10)
α (°)	82.122(2)	81.978(4)	90
β (°)	86.845(2)	75.842(5)	90.3780(10)
γ (°)	88.969(2)	87.649(4)	90
V (Å^3^)	449.980(16)	445.97(4)	1783.47(3)
Z value	1	1	4
density (g/cm^3^)	1.559	1.573	1.573
R1 (final)	0.0326	0.0421	0.0310
wR2 (final)	0.0889	0.1177	0.0815
R1 (all)	0.0329	0.0438	0.0325
wR2 (all)	0.0892	0.1192	0.0826

## Data Availability

Not applicable.

## Supplementary Materials

The following supporting information can be downloaded at: https://www.mdpi.com/article/10.3390/ma15207247/s1, Figures S1, S3, and S5: ^1^H NMR spectra of **4**–**6**; Figures S2, S4, and S6: ^1^H NMR DOSY spectra of **4**–**6**; Figure S7: FTIR spectra of the microcrystals of **4**–**6**; Figure S8: TGA results of the microcrystals of **4**–**6**; Figure S9: simulated powder XRD patterns of **4**–**6** based on their single-crystal data; Table S1: photophysics data of microcrystals of **4**–**6**; Figure S10: lifetime decay curves for microcrystals of **4**–**6**; Figure S11: TDDFT calculation results of [**1**-H_2_]^2+^, [**2**-H_2_]^2+^, and [**3**-H_2_]^2+^; Figure S12: schematic demonstration of the experimental setup for polarized luminescence characterization for a single microcrystal.
