# Supplementary material for: Molecular Cocrystals with Hydrogen-Bonded Polymeric Structures and Polarized Luminescence
Source: Materials (Basel). 2022 Oct 17;15(20):7247. doi: 10.3390/ma15207247 (PMC9609709; doi:10.3390/ma15207247)
Supplement: Supplementary file 1 [file materials-15-07247-s001.zip › materials-1939800-supplementary.pdf]

# Supporting Information

# Molecular Cocrystals with Hydrogen-Bonded Polymeric Structures and Polarized Luminescence

Jing-Yi Zhao [1,2], Fa-Feng Xu [1,*], Zhong-Qiu Li [1], Zhong-Liang Gong [1], Yu-Wu Zhong [1,2,*], and Jiannian Yao [1,2,*]

[1]Beijing National Laboratory for Molecular Sciences, CAS Key Laboratory of Photochemistry, CAS Research/Education Center for Excellence in Molecular Sciences, Institute of Chemistry, Chinese Academy of Sciences, Beijing 100190, China

[2]School of Chemical Sciences, University of Chinese Academy of Sciences, Beijing 100049, China

[*]E-mail: xufafeng@iccas.ac.cn; zhongyuwu@iccas.ac.cn; jnyao@iccas.ac.cn

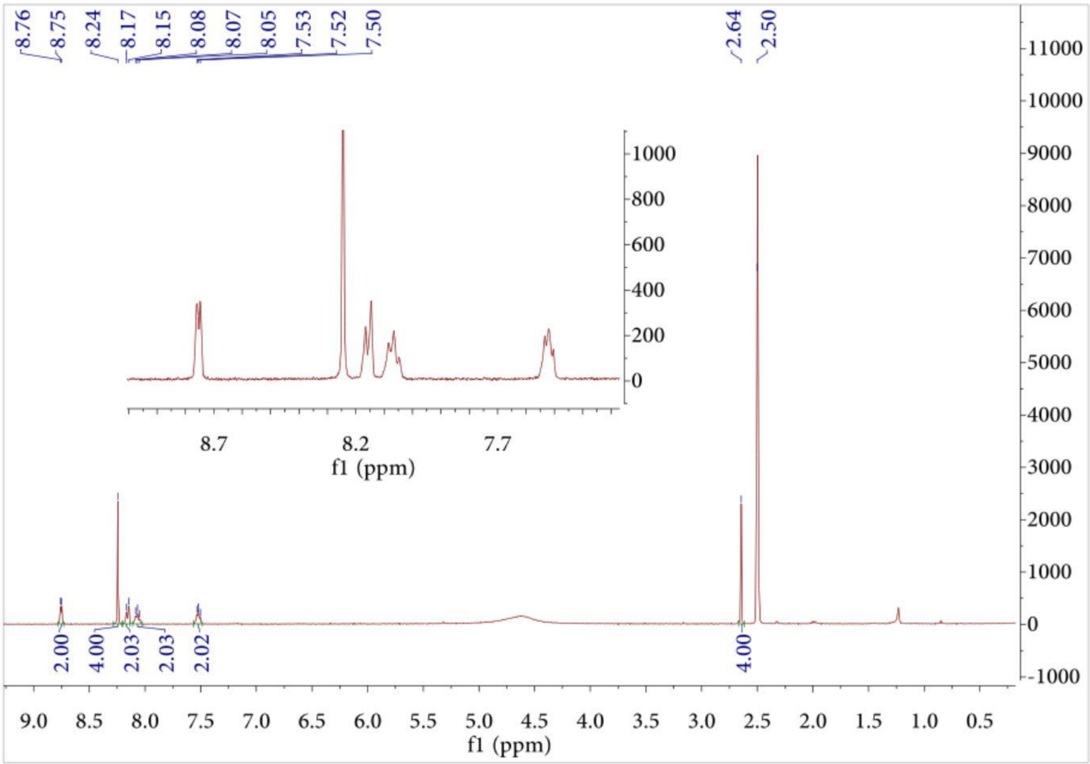


**Figure S1.** $^{1}H$ NMR spectrum of **4** in DMSO-$d_6$.

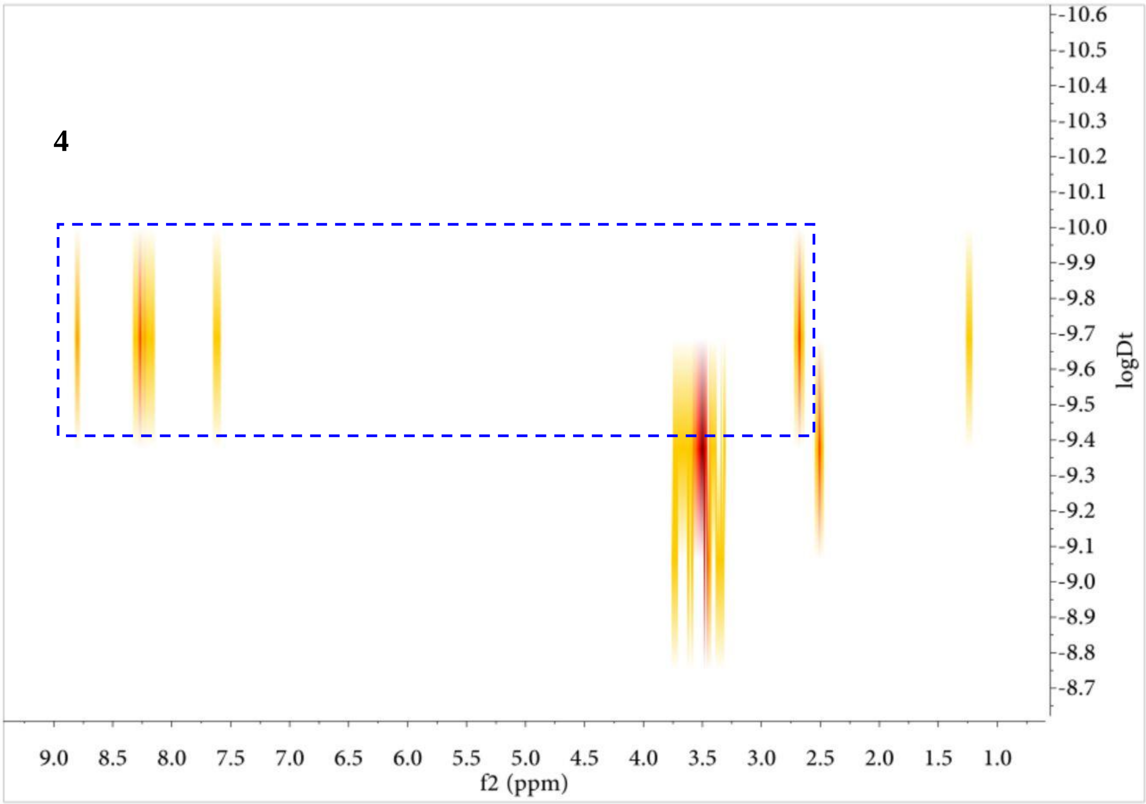


**Figure S2.** $^{1}H$ DOSY NMR spectrum of **4** in DMSO-$d_6$.

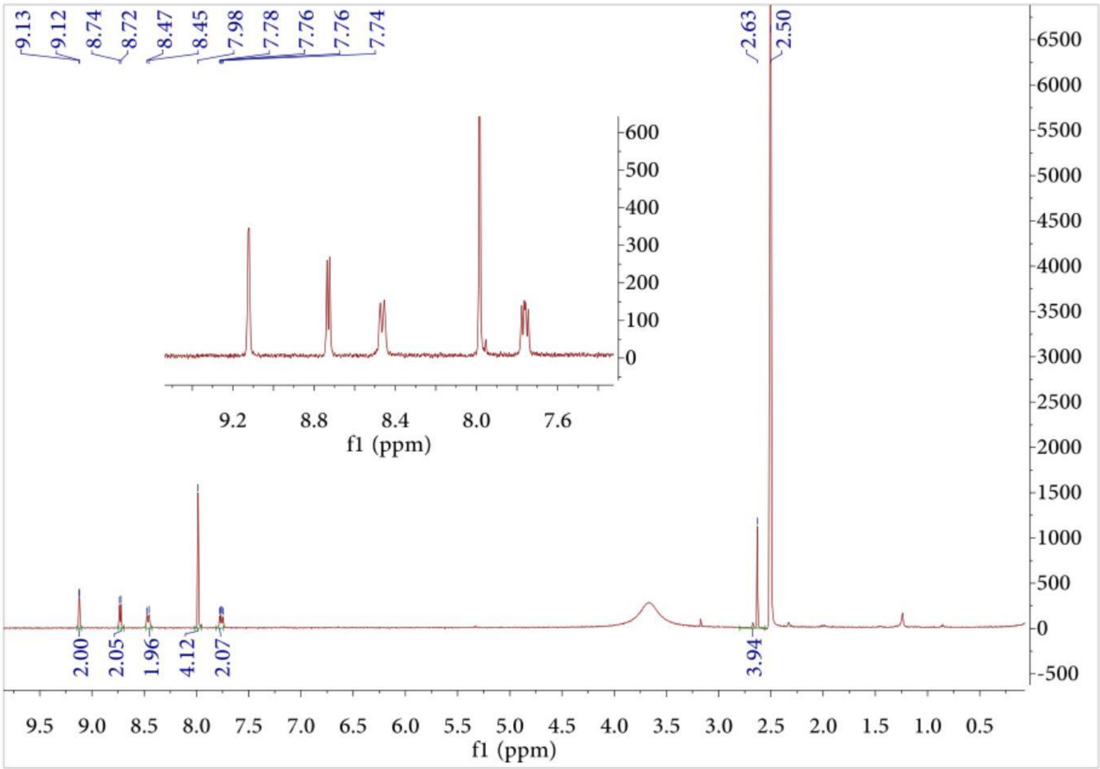


**Figure S3.** $^{1}H$ NMR spectrum of **5** in DMSO-$d_6$.

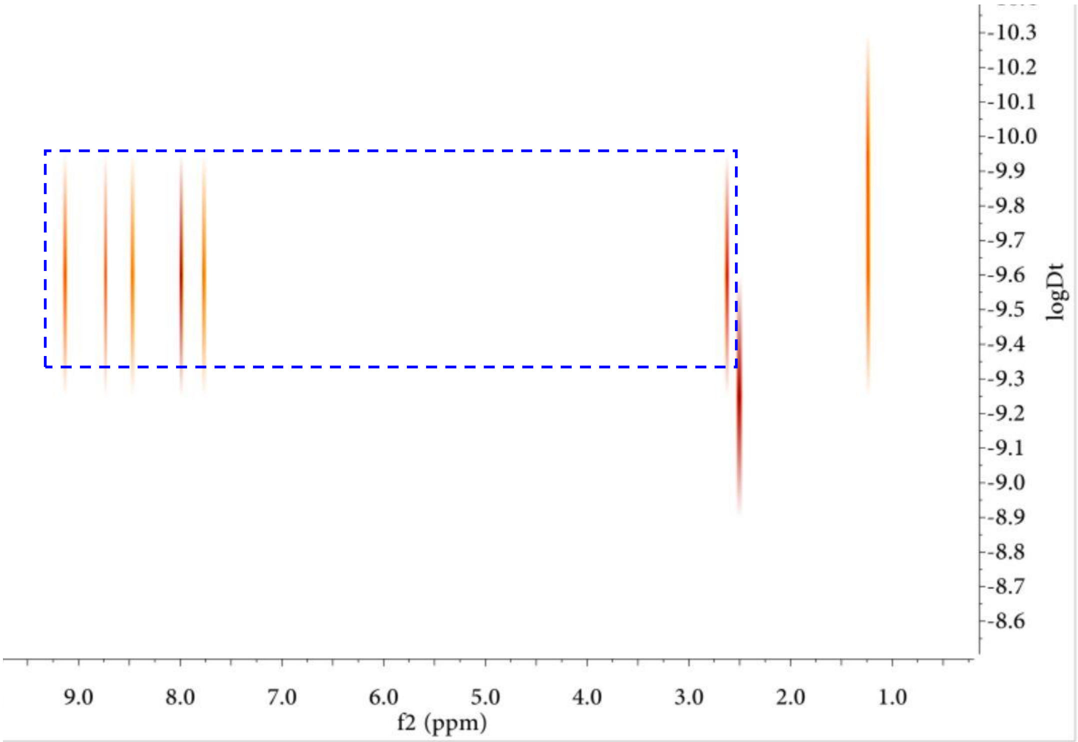


**Figure S4.** $^{1}H$ DOSY NMR spectrum of **5** in DMSO-$d_6$.

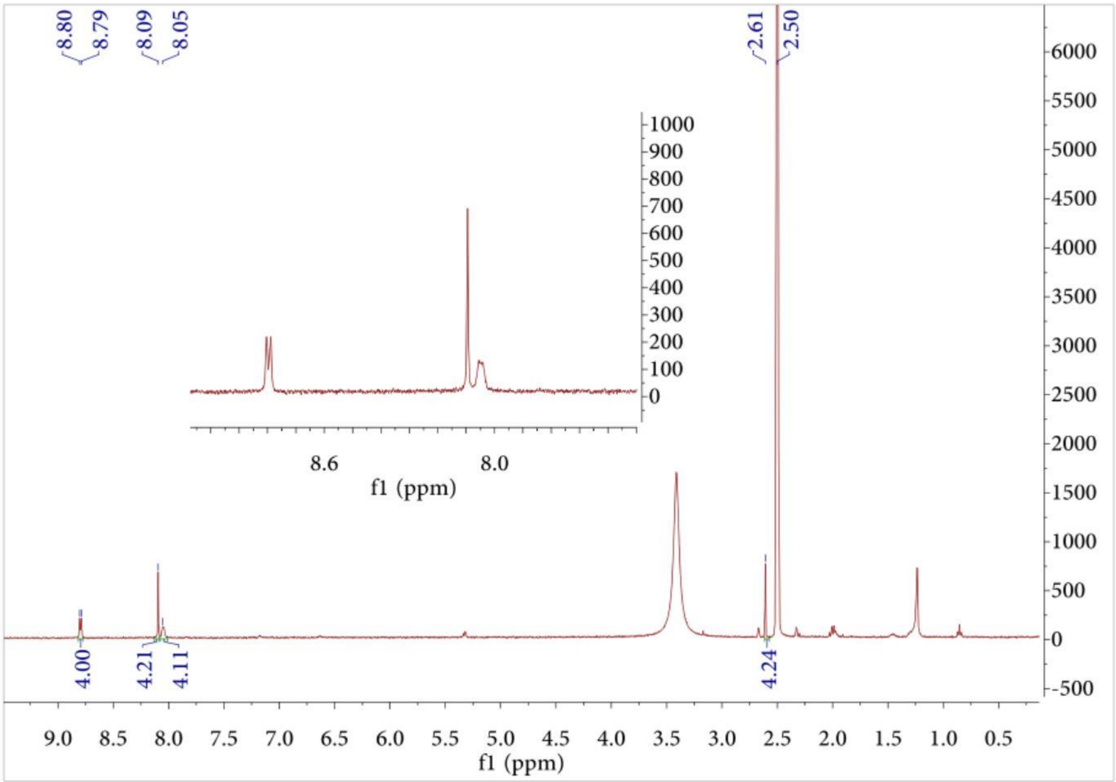


**Figure S5.** $^{1}$H NMR spectrum of **6** in DMSO-d6.

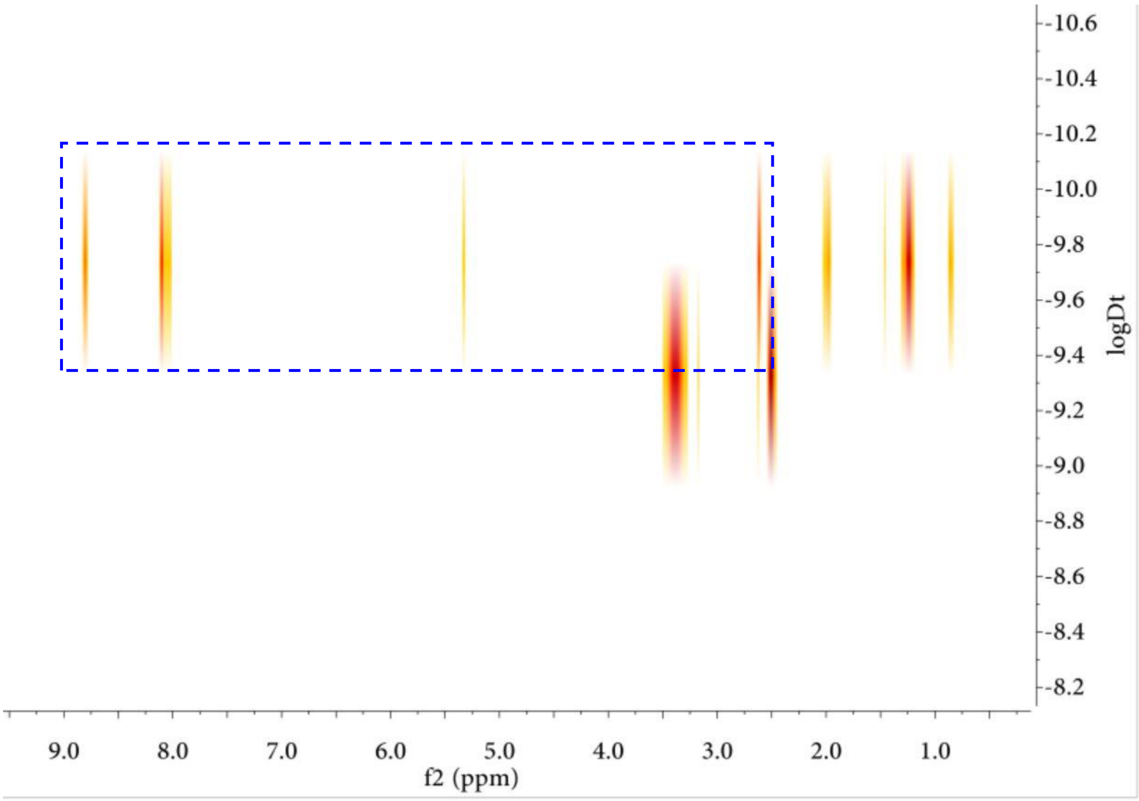


**Figure S6.** $^{1}$H DOSY NMR spectrum of **6** in DMSO-d6.

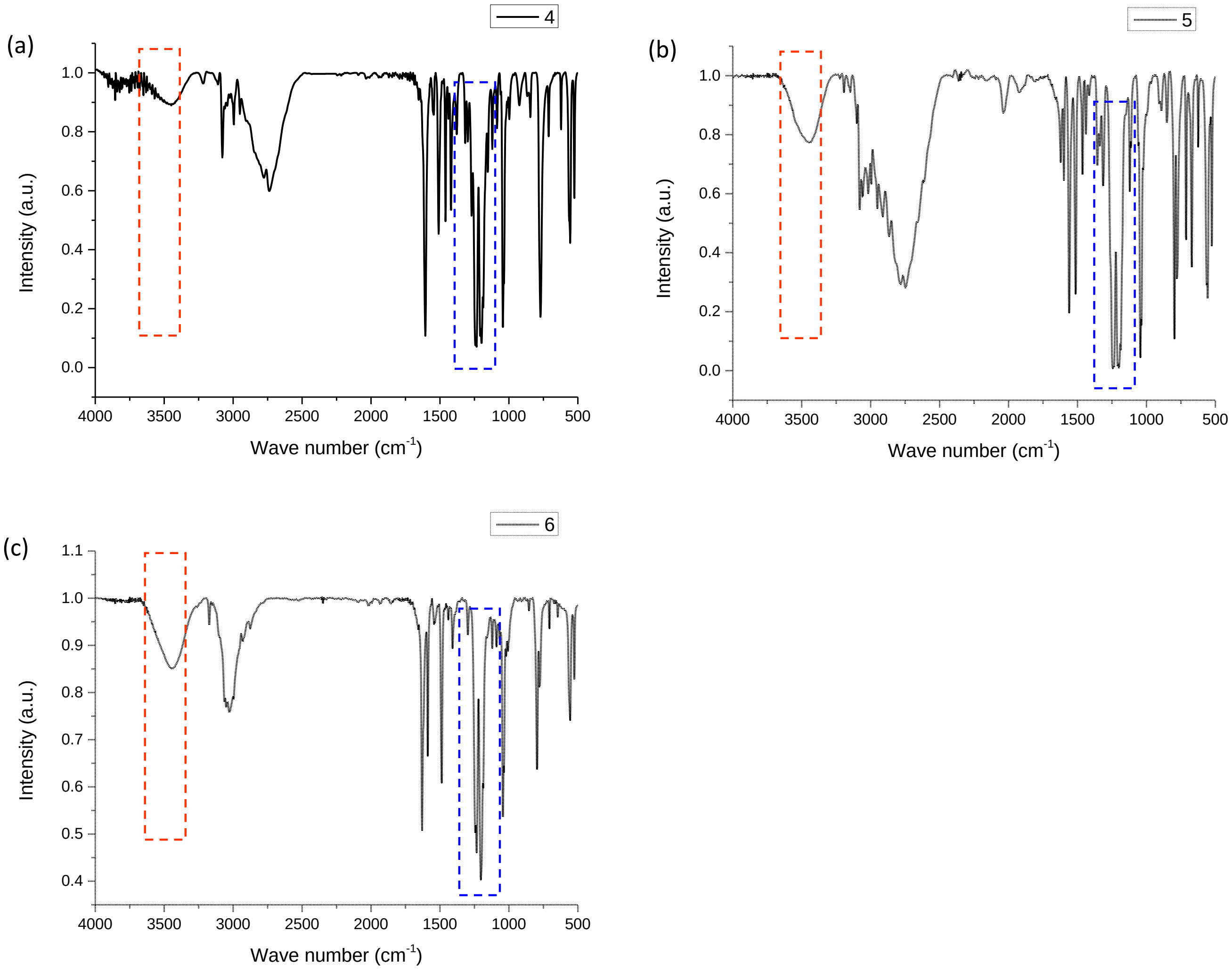


**Figure S7.** FTIR spectrum of the microcrystals of (a) **4**, (b) **5**, and (c) **6** as KBr pellet. The bands at around 3500 cm$^{-1}$ are assigned to the vibrations of hydrogen bonds. The two bands at around 1200 cm$^{-1}$ are ascribed to the symmetric and asymmetric stretching vibrations of the sulfonate groups.

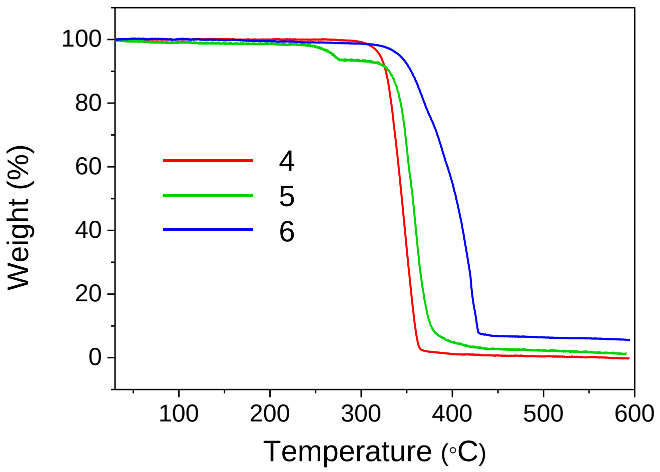


**Figure S8.** Thermogravimetric analysis (TGA) of the microcrystals of **4**, **5**, and **6** with a temperature rate of 10 °C /min in a nitrogen environment (200 mL/min).

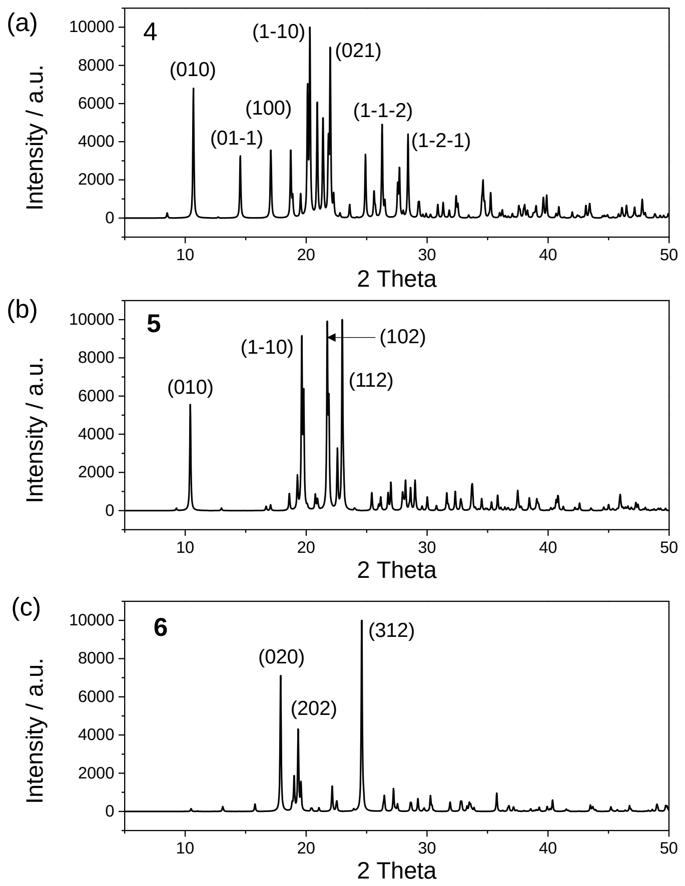


**Figure S9.** Simulated powder XRD patterns of (a) **4**, (b) **5**, and (c) **6** based on their single-crystal data.

**Table S1.** Photophysics data of microcrystals of **4** – **6**.[a]

| Compound | $\lambda_{emi}$/nm | $\Phi$/% | $\tau$/ns | $k_r/10^7\ s^{-1}$ | $k_{nr}/10^7\ s^{-1}$ |
|---|---|---|---|---|---|
| **4** | 385 | 12.9 | 4.8 | 2.67 | 18.03 |
| **5** | 456 | 27.7 | 11.7 | 2.38 | 6.20 |
| **6** | 466 | 18.0 | 1.8 | 9.94 | 45.30 |

[a] $\Phi$ is absolute quantum yield. $\tau$ is averaged life time. See further details in Figure S2. $k_r = \Phi/\tau$; $k_{nr} = (1 - \Phi)/\tau$.

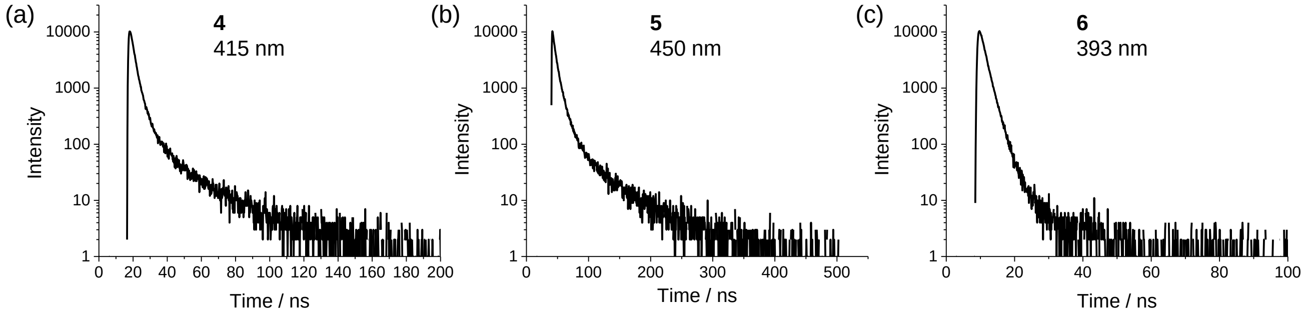


**Figure S10**. Lifetime decay curves for microcrystals of (a) **4**, (b) **5**, and (c) **6**.

Decay curves for microcrystals of **4**, and **5** are tri-exponentially fitted and the decay of **6** is bi-exponentially fitted. The average lifetimes ($\tau_{ave}$) are reported in Table S1, which are determined by $\tau_{ave} = \alpha_1\tau_1 + \alpha_2\tau_2 + \alpha_3\tau_3$.

For microcrystals of **4**, $\tau_{ave}$ = 4.83 ns, $\tau_1$ = 2.27 ns, $\tau_2$ = 6.56 ns, $\tau_3$ = 24.11 ns, $\alpha_1$ = 74.9%, $\alpha_2$ = 16.7%, $\alpha_3$ = 8.4%;

for microcrystals of **5**, $\tau_{ave}$ = 11.66 ns, $\tau_1$ = 3.72 ns, $\tau_2$ = 8.70 ns, $\tau_3$ = 42.34 ns, $\alpha_1$ = 32.0%, $\alpha_2$ = 54.4%, $\alpha_3$ = 13.6%;

for microcrystals of **6**, $\tau_{ave}$ = 1.81 ns, $\tau_1$ = 1.53 ns, $\tau_2$ = 3.47 ns, $\alpha_1$ = 85.8%, $\alpha_2$ = 14.2%.

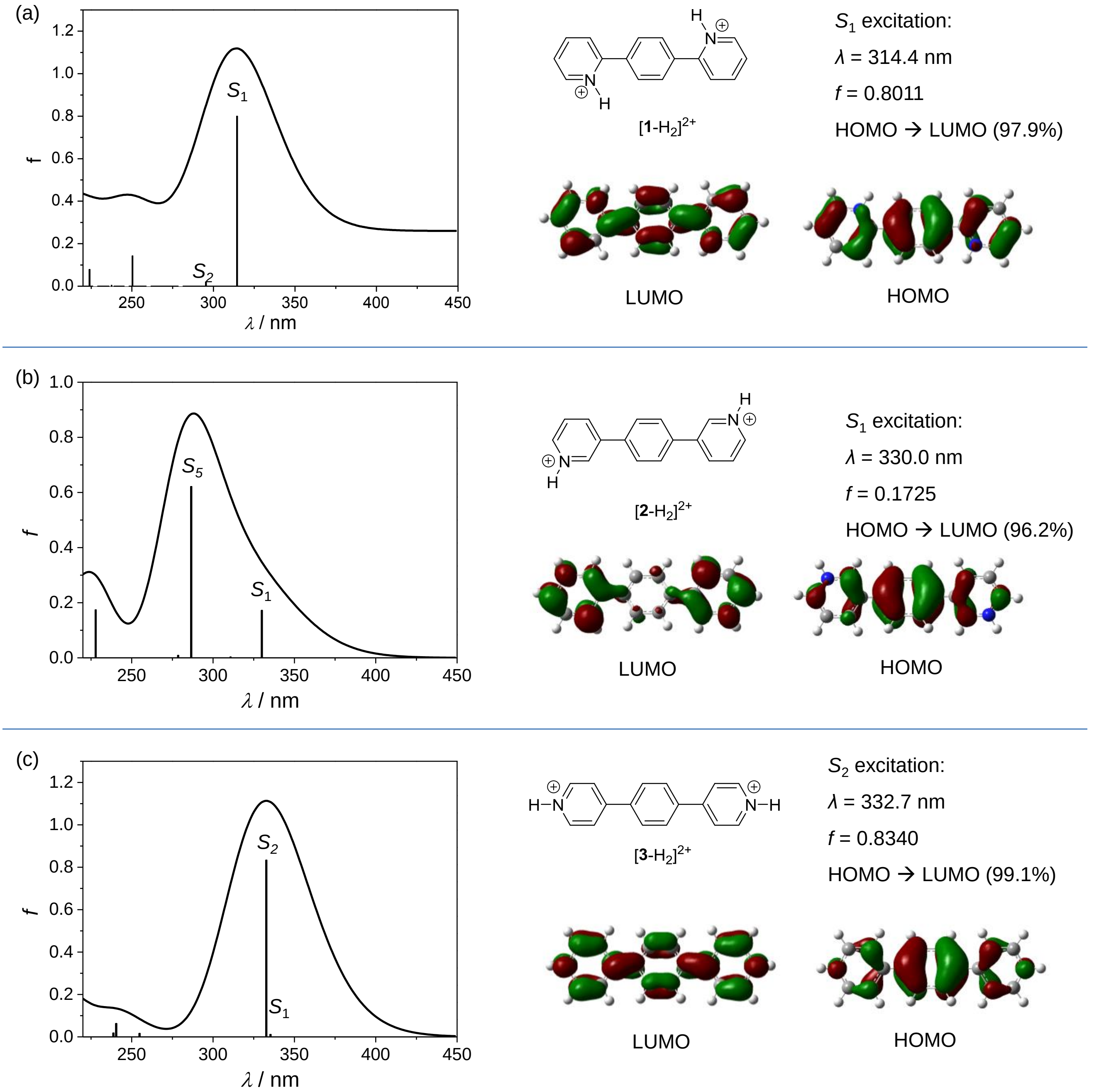


**Figure S11.** TDDFT calculation results of (a) [**1**-$H_2$]$^{2+}$, (b) [**2**-$H_2$]$^{2+}$, and (c) [**3**-$H_2$]$^{2+}$. Left: TDDFT-predicted vertical excitations. Right: chemical structures, DFT-calculated frontier orbitals, and the calculated wavelength (*λ*), oscillator strength (*f*), and percent contribution for the HOMO→LUMO excitation.

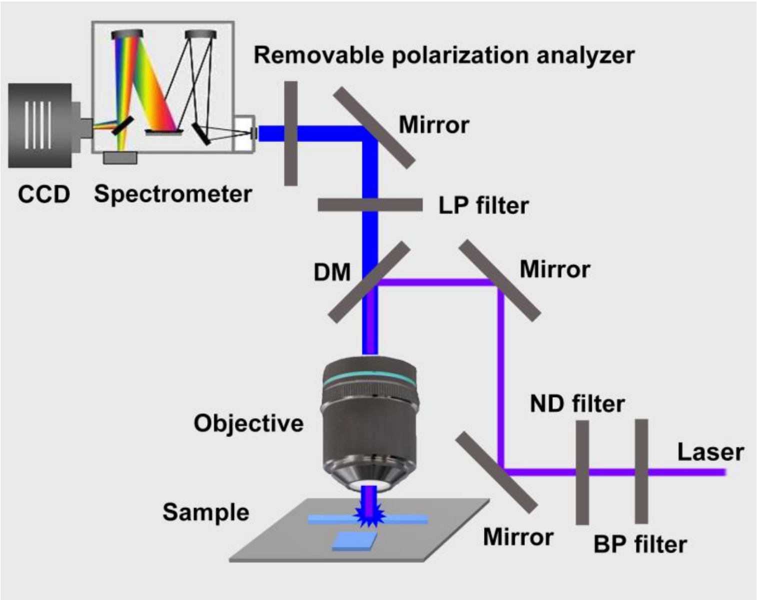


**Figure S12**. Schematic demonstration of the experimental setup for polarized luminescence characterization for a single microcrystal. CCD: charge coupled device; LP filter: long-pass filter; DM: dichromic mirror; ND filter: neutral density filter; BP filter: band-pass filter. The polarization luminescence information of the microcrystal is probed by a removable polarization analyzer plate.

The excitation laser beam (CW, 405 nm) was filtered with a 405/10 nm band-pass filter and then focused down to a 10-μm diameter spot through an objective lens (Olympus M Plan, × 20, N.A. = 0.4) as a nearly uniform pump source. The power at the input was altered by a neutral density filter. The emissions from individual microcrystals were collected by the same objective with a back-scattering configuration and analyzed by Prinston Instrument HRS-300S spectrometer with a thermoelectric-cooled PIXIS 256BR CCD after removing the excitation beam with a 420-nm long-pass filter. The removable polarization analyzer was used before the spectrometer when collecting polarized luminescent spectra of the microcrystals.
